# Medical and Literary Weekly

**Published:** 1859-04

**Authors:** V. H. Taliaferro, A. G. Thomas


### MEDICAL AND LITERARY WEEKLY.

We desire to call attention to the prospectus below, of the Medical and Literary Weekly proposed to be published in this city, by Drs. Taliaferro & Thomas. The gentlemen who have undertaken this enterprise, have talents and scientific attainments, and are certainly about to exert them in a good cause, in which we wish them great success.

We have long most earnestly desired that the nonmedical public could be induced to acquire a sufficient knowledge of the laws of health, to protect itself against the ignorance and infamous pretension, embodied in the almost innumerable forms of empiricism, with which the land is covered at the present day. We invoke the aid of the profession throughout the United States, to the support of the enterprise.

---

*Prospectus of the Medical & Literary Weekly.*—The undersigned propose to publish a weekly paper in the city of Atlanta, Georgia, bearing the above title, the first number of which will be issued on Thursday, May 7th, 1859. The special object in view, is to expose *Quackery* in all its forms, and to show the *impropriety* of using *nostrums* under any circumstances whatever. They will attempt to lay down and explain the Laws of Health as founded in pure Medical Science; and whilst they acknowledge the impossibility of teaching the masses how to treat disease, they hope to be able to do much good, both in instructing them in the operations of the Laws of *Hygiene*, and in warning them against the untold injury which results from the pretensions of *Quacks*, and their ruinous nostrums. They will also furnish their patrons with the most important *Items* of the general News of the times, and promise to devote themselves to the building up of a pure and Scientific Literature. Terms—$2 per annum, in advance.

☞ All letters or communications should be addressed to Editors Medical and Literary Weekly, Atlanta, Ga.

V. H. Taliaferro, M. D.,
A. G. Thomas, M. D.

